# Occupational contact dermatitis from protein in sea products: who is the most affected, the fisherman or the chef?

**DOI:** 10.1186/s12995-017-0150-0

**Published:** 2017-02-10

**Authors:** B. Loddé, P. Cros, A. M. Roguedas-Contios, R. Pougnet, D. Lucas, JD. Dewitte, L. Misery

**Affiliations:** 1 Université Européenne de Bretagne, Rennes, France; 2 Université de Brest, EA 4686 – CS 93837 – 29238, Brest Cedex 3, France; 3 Service de Santé au Travail et Maladies liées à l’environnement, CHRU Morvan, 2 avenue FOCH, Brest Cedex, 29609 France; 4 Service de dermato-vénéréologie CHRU Morvan, 2 avenue FOCH, Brest Cedex, 29609 France; 5 Société Française de Médecine Maritime, 22, Avenue Camille Desmoulins, Brest, 29200 France

**Keywords:** Maritime, Seafood, Contact dermatitis

## Abstract

**Background:**

Protein contact dermatitis has frequently been reported in case studies (usually in cases involving contact with seafood products), but there are very few descriptive series.

The objectives of this present study were firstly to determine the incidence of protein contact dermatitis among fishermen in France and compare it with data from onshore work involving seafood exposure. Second, to discover what factors could explain any differences.

In order to answer these questions we analysed data from the French national occupational disease surveillance and prevention network (RNV3P) and occupational diseases declared to the French National Network for Monitoring and Prevention of Occupational Disease. This retrospective study was done for a 13 year period.

**Case presentation:**

Between 2000 and 2012, we only found eight cases of protein contact dermatitis in the French network. There were no cases of protein contact dermatitis in the seafaring population. The eight cases from the French network are essentially allergies to different fish and chefs are the professionals most affected. Atopy is present in half of these cases.

In the seafaring population we found several cases of allergic delayed-time contact dermatitis due to bryozoans and to gloves but no protein contact dermatitis.

**Conclusions:**

Chefs who have to cook seafood are more at risk of occupational protein contact dermatitis than fishermen. We think that skin protection (that is to say glove wearing) is better implemented in the fishing sector than in the catering profession on shore in France.

## Bakcground

Protein contact dermatitis is an allergic dermatitis first described in 1976 by Hjorth and Roed-Petersen [1]. It is likely to be under-recorded, as the diagnosis requires the association of the presence of specific clinical signs (which can appear in different forms) with a protein-type trigger factor examined by a specific dermato-allergological evaluation.

Its physiopathology involves a type 1 allergic reaction mediated by IgE, and in some cases an associated delayed type 4 hypersensitivity reaction (according to the Gell and Coombs classification) [2]. The delayed reaction is the subject of controversy, however, because of the frequent negative results of delayed-reading tests and the clinical symptoms that occur immediately following contact with the allergen [3, 4]. Hence, the reaction is mainly considered as an immediate hypersensitivity reaction caused by high molecular weight proteins penetrating the epidermis. This penetration is facilitated by the alteration of the epidermis due to, for example, previous atopic dermatitis or irritant contact dermatitis [5–7].

Protein contact dermatitis is characterised by clinical lesions resembling chronic or recurrent eczema. There may be exacerbations in the form of an urticarial or blistering rash that becomes itchy after contact with a product containing proteins. The lesions are located in the contact areas, hence often on the hands but also extending up to the wrists and forearms [5, 7]. There can be non-cutaneous signs, especially among atopic subjects (respiratory signs indicating rhinitis or associated asthma). There are rarely digestive signs when the allergen [2] is ingested, and there are very rarely any systemic reactions [8].

These manifestations are often triggered by contact with plant or animal proteins present in food. When the exposure is intense, which is to say repeated, they can often be considered to be an occupational disease [7]. Protein contact dermatitis was first described in this way in 1976 in sandwich preparers [1].

In France, where employees are compensated in cases of confirmed occupational diseases, the frequency of occupational allergic contact dermatitis is of the order of 7 to 8 cases for 10,000 employees/year, where over 90% are eczemas [5].

From an etiologic standpoint, cases of protein contact dermatitis have frequently been reported in case studies (namely in cases where there is contact with seafood products), but there are very few descriptive series [9, 10]. Hence, we sought to determine the frequency of these specific cases of dermatitis amongst those in professions where they are most likely to handle such products.

Being at the start of the distribution chain, professional fishermen seemed, at first glance, to be the group most exposed to such proteins.

The main objective of this study was to determine the frequency of protein contact dermatitis linked to seafood products within the group of the working population that appeared to be the most exposed.

The secondary objectives were to compare the data obtained with data from cases reported during professional activities on land, to research differences between them and to determine what factors could explain these.

In order to carry out this study, which was both descriptive and retrospective, we selected two populations in which we could find indicators of occupational pathologies. The first group consisted of a population of professional French fishermen, and the other of all those who had sought medical advice at a French centre for occupational diseases. There are 31 of these centres in teaching hospitals spread across the nation.

All of the patient consultation records obtained from these occupational disease centres, which are specialised units in hospitals, are entered and coded by senior doctors in the French National Network for Monitoring and Prevention of Occupational Disease (RNVPPP) [11]. This network identifies all the occupational health problems that have been referred for expert advice within a hospital centre. The resulting files are often complex as they describe cases that have not been resolved by an urban general medicine practice.

For each case, the disease code (the International Classification of Diseases, 10th edition), the (occupational) substance, company’s activity, and patient’s occupation are recorded during the consultation. The case record is concluded by a clinical summary.

This allowed us to research, within the RNVPPP, all of the cases in which the disease code (CIM-10) corresponded to an allergic contact dermatitis and for which the substance code was fish, crustacean, mollusc or seaweed. We then asked each centre where the cases had been coded for the patients’ medical files in order to analyse them. These files were then anonymised and archived. A file had to contain at least one detailed history of the disease, one clinical exam, and one allergological evaluation done by means of a skin prick test and/or a patch test and/or specific IgE testing. The patients had to have seen a senior doctor in order to establish the diagnosis. The patients with typical delayed-time allergic contact dermatitis and/or irritant contact dermatitis (related to soaps, detergents, etc.), or imprecise or questionable clinical histories were excluded from our study. The focus of the study was on occupational protein contact dermatitis.

For comparison, we also researched all cases of occupational skin disease declared under the ENIM (the social security scheme for maritime professions). This retrospective national study focused on illnesses reported over a 13-year period. The files were analysed by ENIM, which compensates professional seafarers who are officially affected by an occupational maritime disease.

## Case presentation

Among the 145,293 medical cases registered in the RNVPP between 2000 and 2012, 8 patients were declared as being affected by protein contact dermatitis caused by fish or crustaceans or sea product. Moreover 2 other cases appeared imprecise and seemed to be irritant contact dermatitis to sea products (and to other irritants). Because there was no mention of positive prick-test or positive IgE these 2 cases were excluded from our description.

There were no reported cases of protein contact dermatitis in the population of seafarers (there being an average of about 35,000 registered seafarers per year). Within this population, 25 cases of occupational skin disease caused by allergies were found in 13 years. These were all cases of typical allergic delayed-time contact dermatitis. The allergens were mercaptobenzothiazole, thiuram (a component of protective gloves), bryozoans, and even seaweeds, but there was no mention of occupational dermatitis caused by fish or crustacean proteins.

### Description of the eight cases of occupational protein contact dermatitis

Seventy-five percent of patients (*n* = 6) were men. The average age at the time of diagnosis was 26 years (range = 19–50 years). The latency of the diagnosis was, on average, 36 months, with significant deviations ranging from 4 months to 13 years.

Seven out of the eight patients were chefs or worked in the catering sector (that is to say about 900, 000 employees (with seasonable variations) per year in France, 200, 000 of whom are chefs); one patient was a pet shop employee. Four patients had a prior history of skin problems (eczema) and one patient was asthmatic (Table 1).

**Table 1:** Description of the population affected by seafood-based protein contact dermatitis

Case	Age	Sex	Occupation	Diagnostic latency	Atopy	Allergen positive testing	Allergen negative testing
1	50	male	chef	14 months	+	Salmon	Crustaceans, trout, pollock, whiting, hake, seabass, scallop
2	23	male	chef	3 years	+	Salmon, whiting	Crustaceans, hake, pollock, scallop
3	19	female	chef	4 months	-	Pollock	Shrimp, salmon, whiting, hake, scallop
4	20	male	chef	6 months	+	Crustaceans	Pollock, whiting, hake, seabass, scallop
5	25	male	chef	4 months	-	Salmon	Crustaceans, trout, pollock, whiting, hake, seabass, scallop
6	33	male	chef	13 years	-	Salmon, monkfish, scallops	Shrimp, trout, pollock, whiting, hake, seabass,
7	19	male	chef	-	+	Salmon	Crustaceans, trout, pollock, whiting, hake, seabass, scallop
8	21	female	Pet shop employee	7 months	-	Daphnia	Shrimp, food for fish

In every case, the diagnosis was made by a senior doctor and included a skin prick test. The pricks were made with extracts of foods usually handled for each patient (food extracts were prepared by cutting pieces of fish or crustacean flesh which were then crushed into a solution with physiologic serum then put into contact with patient skin to be pricked through), and also with commercial extracts for five foods (salmon, hake, bass, shrimp and lobster).

For negative control prick test, physiologic serum was used. For positive control prick test, 9% concentration of codeine phosphate solution was used. These solutions and commercial extracts of fish allergens solutions can be purchased at ALK® laboratories (55 271 Varennes en Argonne Cedex, France).

The mean-size of the positive control test was 2 mm for the wheal and 30 mm for the erythema (0/0 for the negative one). A positive test was at least the same size as the wheal of the positive control test with at least the same size of the erythema in comparison with the positive control test.

Reactions to salmon were the most frequent. They were reported for five patients (Table 1).

Patch testing was also carried out for these 8 patients. The patch test procedure was the same for all of them. Concerning patch tests, 8 mm diameter aluminium round finn chambers on Scanpor were used to perform the exploration. In France these devices are essentially provided by Stallergenes® (6, rue Alexis de Tocqueville 92160 Antony, France). Stallergenes® is commercializing the products of SmartPractice® 3400 E. McDowell Rd, Phoenix, AZ 850008 USA.

At the time of second appointment (48 h later) patch tests were removed and third appointment was given 96 h later. There were Standard European batteries, which showed sensitivity to nickel in one case. The patch tests carried out with the fish and crustacean flesh were prepared by cutting little pieces of native sea product and put in the chamber. They were all negative.

No scratch tests were performed, and none of the specific IgE testing came back positive for proteins of any type (parvalbumin) or for any species of fish. The concurrence of the clinical history and positivity of the skin prick tests hence confirmed the diagnosis.

None of the patients showed local or systemic reactions during ingestion of the substances in question for seafood-based protein dermatitis. No urticarial reaction to seafood were reported by the patients. By contrast, four out of eight patients reported skin lesions that had been diagnosed as irritant contact dermatitis in the preceding months.

Finally, none of the eight patients mentioned using gloves when handling fish.

## Discussion

The risk of occupational seafood-based protein contact dermatitis appears to be low in the populations that we studied. It is therefore difficult to draw statistical conclusions with so few occurrences.

The social security agency for professional seafarers has no recorded cases of protein contact dermatitis. Within this population, the occurrence of occupational allergic dermatitis entitling the sufferer to compensation is also low since, over the last 13 years, reports of occupational dermatological diseases have only occurred 0 to 5 times per year (25 in 13 years). These were always related to allergic contact dermatitis based on a delayed-type contact allergy rather than being related to protein contact dermatitis [12–14].

The simplest explanation could be that the disease did not occur during the study period (13 years), although a number of articles do in fact indicate a low prevalence of this disease. Moreover, having been described for the first time in 1976, protein contact dermatitis is still a little-known disease and is hence probably under-diagnosed [1, 15]. Additionally, our study is retrospective as it uses the registry for the compensation of occupational diseases. The registry is non-exhaustive in its overview of skin diseases present in the fishing profession (infectious, cancerous and other non-allergic skin diseases having been left out of the search). As such, the study selects only the patients who chose to have their skin problem diagnosed as well as to claim for compensation.

It is indeed possible that some fishermen, aware of their condition, hide their dermatitis out of fear of the seafarer’s doctor revoking their medical clearance to embark. It is therefore possible that the fear of losing one’s job (for medical reasons) may often make people reluctant to talk about their health problems with their regular doctor, and even more so with the doctor in charge of determining their medical fitness for work.

In a fish preparation plant, Aasmoe et al. found over 50% of workers had clinical occupational dermatitis but only 2.6% of the employees said they felt hindered by their condition [16]. The study sheds light on the fact that, in a population of workers such as fishermen, who are accustomed to difficult working conditions, there is a relative trivialization of skin problems that are considered minor. Such skin problems are therefore never mentioned to a health professional.

Another explanation that needs to be considered is a healthy worker effect: affected workers may have left the industry of their own accord so as to prevent their health problem from worsening rather than gone to see a doctor about their dermatitis or gone as far as to declare the condition. Their positions are then filled exclusively by healthy individuals. This hypothesis is frequently put forward to explain the rarity of this pathology in occupations that are particularly at risk [16, 17]. Nonetheless, we could also investigate the question of the positive protective influence of the maritime environment with regard to sensitivity to seafood proteins.

Additionally, glove protection may limit exposure sufficiently to avoid triggering sensitivity. In this context, fishermen seem very diligent in wearing skin protection gear while carrying out their professional tasks, during which the risk of injury is also high [12].

Furthermore, atopy, which was frequent in the second population of professionals affected by protein contact dermatitis, is very infrequent in the seafaring population [13]. Certain atopic pathologies, such as unstable atopic asthma, make it difficult for sufferers to do these jobs, resulting in the exclusion of a population at risk of developing protein contact dermatitis.

Among the other people working with seafood, the population of workers in seafood processing companies would logically be expected to be the most affected. Among employees of seafood processing companies in France, however, no cases of protein contact dermatitis were recorded, in contrast with results in a number of publications [16–18]. When compared to major fish-producing countries such as Norway and South Africa, this result is surprising.

As is the case for fishermen, the explanation may be that primary protection, such as wearing gloves and other protective clothing in factories may be sufficiently well applied so as to limit the occurrence of these diseases, in addition to a strict medical aptitude filter, which may exclude the more at-risk workers with atopic symptoms.

Given the employment statistics in France, fish processing jobs are often seasonal ones. Frequently, these professions see a significant turnover in the labour force and a rapid shift in career orientation when an employee isn’t satisfied with the type of work. Kalogermitros showed that the intensity and, more importantly, the duration of exposure are risk factors in fish processing companies, which perhaps explains the absence of cases among French workers since they frequently change jobs or careers [19]. Furthermore, the population of seasonal workers often bypasses occupational medical examinations, and thus workers under-report the occupational diseases that could subsequently be of harm to them.

The population of chefs is the most affected population in our study. They often handle fish bare-handed, which may explain the prevalence of protein contact dermatitis in our study. Moreover, there are other factors that influence the increased risk for protein contact dermatitis. Halkier et al. showed that skin reactions particularly increased with the post-mortem age of the fish [20]. In addition, the liquid from fish, which has a high protein content, has been recognised as a risk factor in several studies [16, 21]. In a study by Aasmoe, those doing jobs involving filleting and (bare-handed) shrimp peeling were the most at risk among 883 employees of a seafood processing plant [16]. Given the preparation techniques, it appears logical that chefs would be the most affected occupation.

With salmon, there may be an added risk of protein contact dermatitis. The question of whether salmonid proteins are more sensitizing than proteins from other ocean fish warrants further investigation. Additionally, because the species can also be fished in fresh water and produced in aquaculture, it may be less often caught by fishermen at sea leading to a lower prevalence of associated skin problems in this population and a greater incidence in other groups.

Ultimately, the rarity of this dermatitis even among at-risk occupations can be explained, as is the case for fishermen, by the specificity of the files from occupational disease centres. Indeed, only the most complicated cases seek medical attention in these hospital centres, leading the population to be made up of all the “worst” cases, which could not be solved in general medical practices. In order to corroborate this information, we can say that over a 6-year period in Nancy (France), four cases of seafood protein contact dermatitis were reported among chefs by means of a local awareness network (both within and between hospitals), while we have seen eight cases throughout France in 13 years, including one chef from Nancy in the RNVPPP [22].

Finally, this skin disease is perhaps under-diagnosed because of the recentness of its description and complex and unresolved aetiology. Many indeed believe that it may be triggered by a combination of type 1 and 4 reactions from the Gell and Coombs classification. Arguments to support this are the existence of positive patch tests with more moderate reactions [23], the positive nature of scratch tests (which allow for a better penetration of the allergen) [24], the efficiency of corticoids as opposed to antihistamines, and the cutaneous symptoms with rare or minimal urticarial reactions [25]. Moreover, we were amazed by the absence in our population, as well as in the literature, of a description of systemic reactions during the ingestion of raw or cooked seafood by affected patients [7]. Only two cases were reported, and both were in patients with a filaggrin mutation, which is a known risk factor for allergic disease [26, 27]. The physiopathology is therefore complex and needs to be clarified in order to gain a better understanding and make a better diagnosis of this pathology.

Nevertheless in our study none of the patients with positive prick test results to seafood had corresponding specific IgE in the serum. Perhaps because the explored allergen is not the same for the prick-test and the specific IgE and/or the time of the blood testing (sometimes very later after the beginning of the disease) was not at the maximum synthesize of these specific IgE.

## Conclusion

Chefs (specifically those who work with seafood) appear to be at higher risk of occupational protein contact dermatitis than fishermen. The influence of glove protection during fishing activities as compared to the bare-handed work of many chefs warrants further investigation. Furthermore, atopy that seems to be a risk factor among chefs is infrequent among fishermen. Indeed, the condition is frequently a reason for medical unfitness for onboard work, especially when the person has already suffered from unstable atopic asthma.

## Abbreviations

CIM-10: Classification Internationale des Maladies 10^ème^ version (International classification of diseases 10th edition)
ENIM: Etablissement National des Invalides de la Marine (the social security scheme for maritime professions)
IgE: Immunoglobulin E
RNV3P: Réseau National de Vigilance et de Prévention des Pathologies Professionnelles (French national occupational disease surveillance and prevention network)
